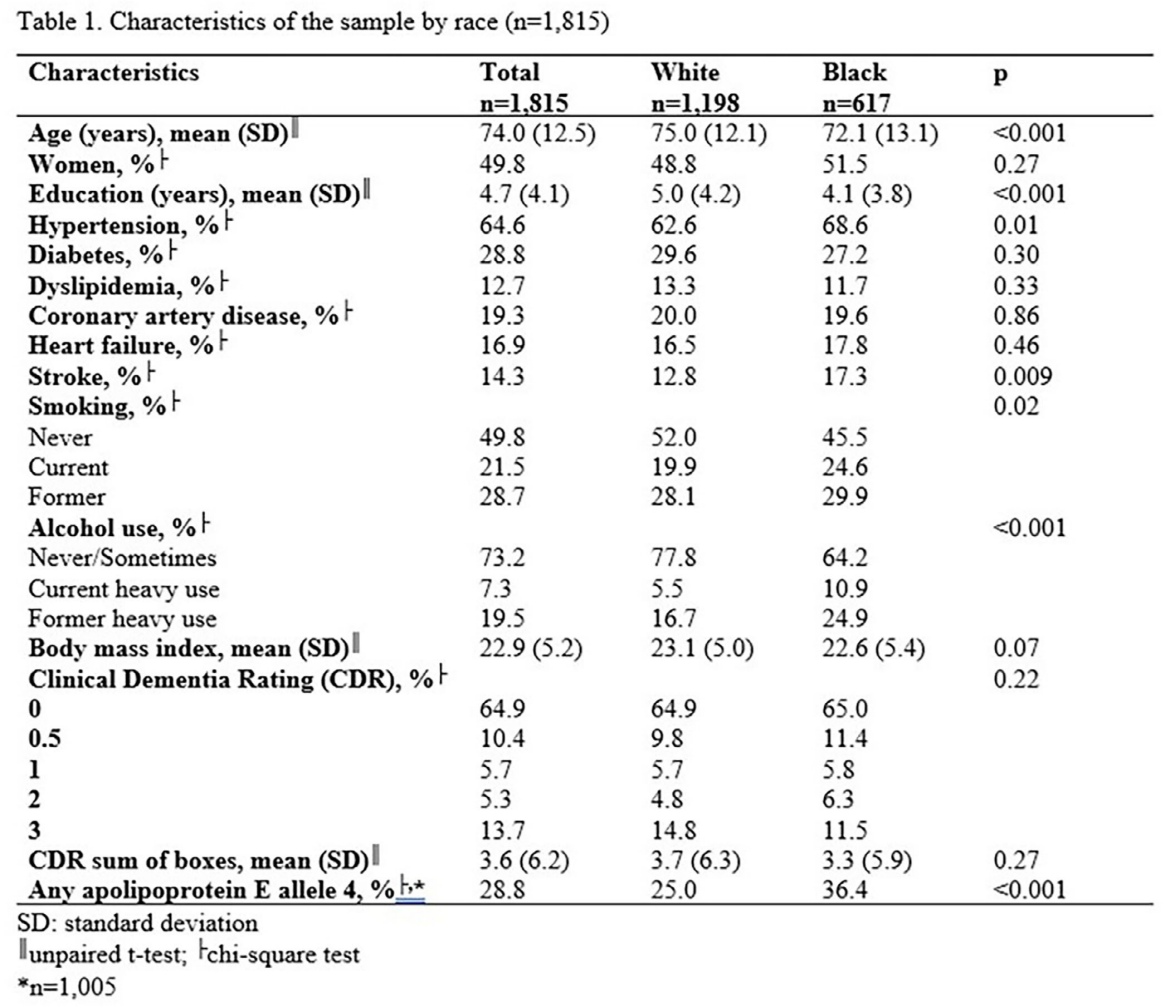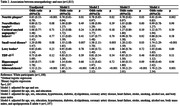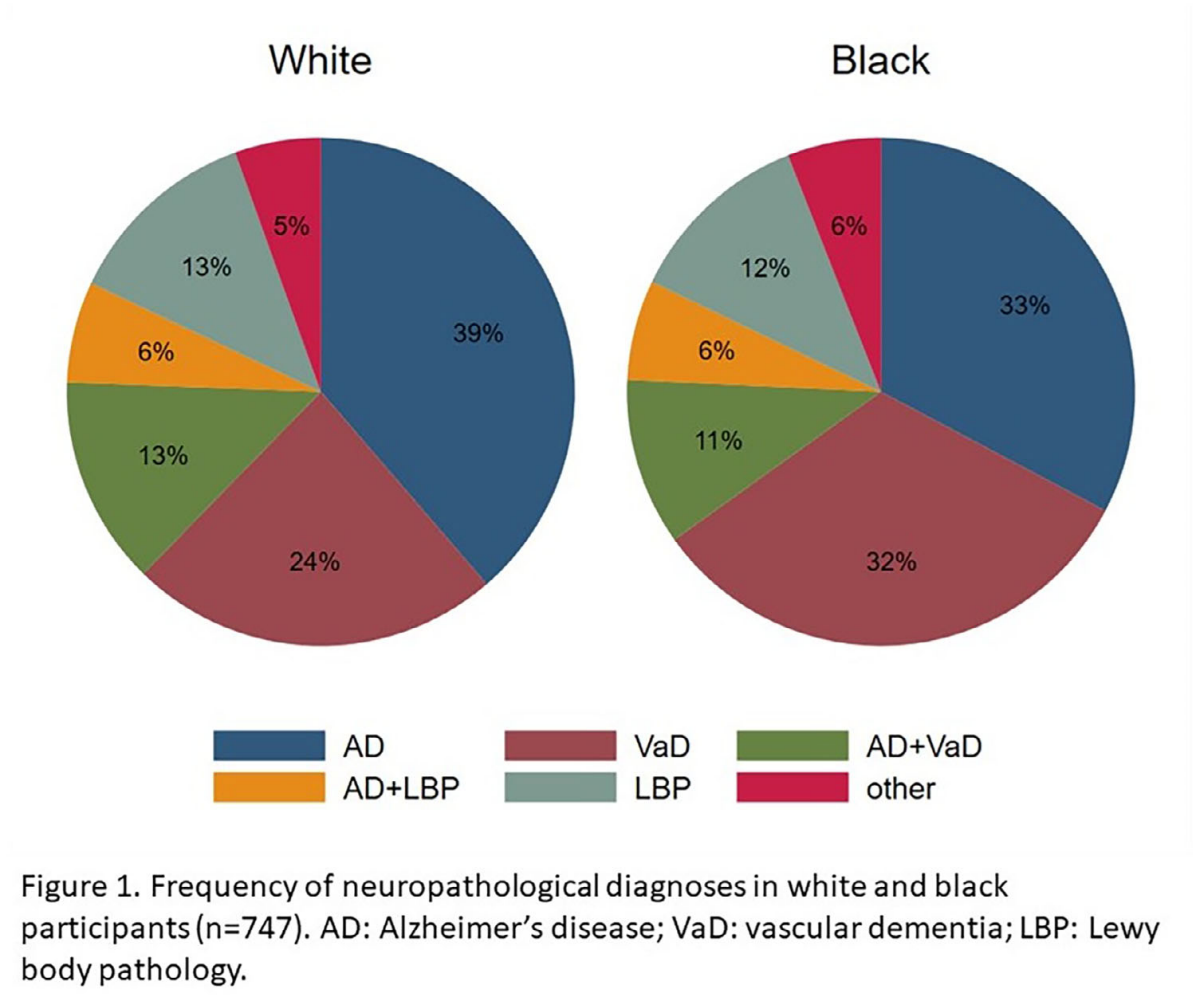# Racial differences in neuropathology and cognitive abilities

**DOI:** 10.1002/alz.090333

**Published:** 2025-01-09

**Authors:** Claudia Kimie Suemoto, Renata Elaine Paraizo Leite, Vitor Ribeiro Paes, Roberta Diehl Rodriguez, Alberto Fernando Oliveira Justo, Michel Satya Naslavsky, Mayana Zatz, Carlos Augusto Pasqualucci, Ricardo Nitrini, Eduardo Ferriolli, Wilson Jacob‐Filho, Lea T. Grinberg

**Affiliations:** ^1^ University of São Paulo Medical School, São Paulo, São Paulo Brazil; ^2^ Division of Geriatrics, Department of Internal Medicine, University of Sao Paulo Medical School, São Paulo, São Paulo Brazil; ^3^ University of São Paulo, São Paulo Brazil; ^4^ UCSF Alzheimer’s Disease Research Center, San Francisco, CA USA; ^5^ Memory and Aging Center, Weill Institute for Neurosciences, University of California, San Francisco (UCSF), San Francisco, CA USA

## Abstract

**Background:**

Racial differences in dementia prevalence and incidence were found with higher dementia burden in African descendants. Previous neuropathological studies were conducted mostly in white participants in convenience samples. Further studies in diverse populations are important to foster the understanding of race differences in dementia pathology. We aimed to compare the frequencies of neuropathological lesions between black and white participants and the association of cognitive abilities and race in an autopsy study.

**Method:**

In a cross‐sectional community‐based autopsy study, samples were collected at the Biobank for Aging Studies from 2004 to 2023. A family member reported the deceased’s race. The frequency of neurodegenerative and cerebrovascular lesions was evaluated using immunohistochemistry and hematoxylin‐eosin staining in 13 selected cerebral areas. We also examined the association between cognitive abilities with the Clinical Dementia Rating Scale and race. Neuropathologists were blinded to cognitive outcomes. To examine the associations of race with neuropathology and cognitive abilities, we used regression models adjusted for age, sex, education, clinical variables, and apolipoprotein E genotyping.

**Result:**

In 1,815 participants, the mean age was 74.0±12.5 years old, 50% were women, 34% were black, the mean education was 4.7±4.1 years, and 35% had cognitive impairment. Small vessel disease (SVD) (OR = 1.74, 95% CI = 1.29‐2.35, p<0.0001), siderocalcinosis (OR = 1.70, 95% CI = 1.23‐2.34, 0.001), and neuropathological comorbidity (OR = 1.35, 95% CI = 1.12‐1.63; p = 0.001) were more frequent in blacks compared to whites, while neuritic plaques were less frequent (OR = 0.61, 95% CI = 0.44‐0.83, p = 0.002). Likewise, AD diagnosis was more frequent in whites (Whites: 39%; Blacks: 33%), while vascular dementia was more common among black participants (Whites: 24%; Blacks: 32%). Race was not associated with cognitive abilities, nor was an effect modifier in the association between neuropathology and cognition.

**Conclusion:**

In a large community‐based autopsy study, AD pathology was more frequent in whites, while vascular pathology and neuropathological comorbidity were more frequent in blacks. Cognitive abilities were similar between race groups, and race was not an effect modifier in the associations of cognition with neuropathologies. Further neuropathological studies in diverse samples are needed to understand race disparities in dementia burden.